# Beyond Killing *Mycobacterium tuberculosis*: Disease Tolerance

**DOI:** 10.3389/fimmu.2018.02976

**Published:** 2018-12-19

**Authors:** Maziar Divangahi, Nargis Khan, Eva Kaufmann

**Affiliations:** Meakins-Christie Laboratories, Departments of Medicine, Microbiology and Immunology, Pathology McGill University, McGill International TB Centre, McGill University Health Centre, Montreal, QC, Canada

**Keywords:** host defense against pathogenic bacteria, disease tolerance, tuberculosis, innate immunity, adaptive immunity

## Abstract

Host defense strategies against infectious diseases are comprised of both host resistance and disease tolerance. Resistance is the ability of the host to prevent invasion or to eliminate the pathogen, while disease tolerance is defined by limiting the collateral tissue damage caused by the pathogen and/or the immune response without exerting direct effects on pathogen growth. Our incomplete understanding of host immunity against tuberculosis (TB) is predominately rooted in our bias toward investigating host resistance. Thus, we must refocus our efforts to understand the entire spectrum of immunity against *M. tuberculosis* to control TB.

## Introduction

Tissue homeostasis is essential for optimal physiological function and overall host fitness for survival (1). Thus, we have evolved with a complex tissue adaptation that involves cellular stress responses and, paradoxically, inflammation to maintain integrity, and functional capacity of an organ despite constant endogenous or exogenous insults, including infections. Historically, the dogma of host defense against infection was unilaterally aimed at eliminating the root of disease (i.e., the pathogen) and ultimately led to the discovery of antimicrobial drugs. While the discovery of antibiotics to directly restrict the growth of pathogens was a “revolution” in medicine, this accelerated drug-induced natural selection leading to the spread of drug-resistant pathogens. Today, the persistence of infectious diseases, the lack of vaccines for major chronic infections (e.g., tuberculosis, malaria, and HIV), as well as the decline in new antibacterial drugs in the pipeline are all indications for the urgent need of novel therapies that require a better fundamental understanding of host defense against infections.

Now, it is increasingly understood that host defense strategies against infectious diseases are comprised of both host resistance and disease tolerance. Host resistance is the ability of the host to prevent invasion or to eliminate the pathogen (2), while disease tolerance is defined by limiting the tissue damage caused by the pathogen and/or the immune response (3). Unlike resistance, disease tolerance does not necessarily exert direct effects on pathogen growth. For this reason, host resistance was considered as the central arm of host defense against infections. In fact, our inconsistency in understanding immunity against infectious diseases might be in part due to our bias toward host resistance to infections. However, this dogma has been recently challenged as we are gaining more fundamental knowledge from simple organisms such as the plant host defense mechanisms (4–7).

As plants are stationary, they have evolved many sophisticated host defense mechanisms to endure severe diseases caused by a large variety of pathogens, including fungi, bacteria, and viruses. In the late 1950s to early 1970s, it was initially observed that plants can tolerate an infection with normal yield without affecting the pathogen load, which was termed “disease tolerance” (2, 8, 9). Most recently, Medzhitov, Schneider, and Soares broadened this concept (10), which has led to a growing appreciation for the crucial role of disease tolerance in invertebrates and vertebrates against infectious diseases (11, 12).

*Mycobacterium tuberculosis* (*Mtb*) has coevolved with humans for 70,000 years (13, 14) and achieved an evolutionary trade-off that infrequently compromises host survival. This trade-off has been conventionally considered to be dependent on host resistance for limiting the growth of *Mtb*. However, our understanding of natural immunity in 90 to 95% of infected individuals who become disease-free is extremely limited. As this latter population constitutes approximately a quarter of the world population (15), it is imperative that we delineate the mechanisms underlying host resistance vs. host tolerance during TB. In this Mini-Review, we focus on recent studies that shed light on the cellular and molecular mechanisms of disease tolerance to *Mtb* and aim to fill this gap in knowledge of immunity against TB.

## Tuberculosis

**Figure 1:**
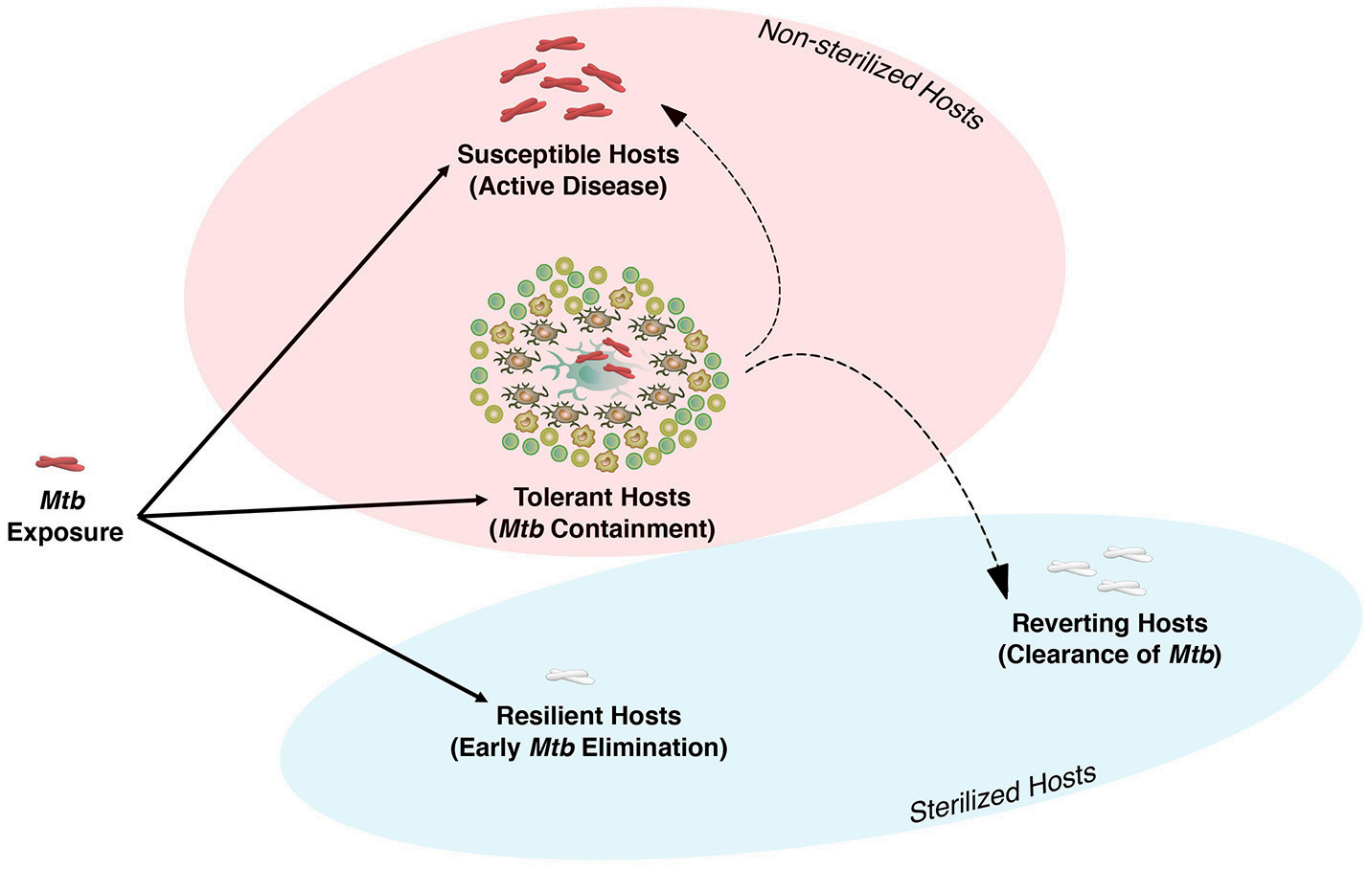
The spectrum of *Mycobacterium tuberculosis* infection in humans. Humans and *Mtb* have co-evolved to reach a dynamic equilibrium. There are three major outcomes following exposure to *Mtb*. (1) Resilient Host: These individuals are able to eliminate the bacteria at the early stage of infection via host defense mechanisms of the upper or lower airways. (2) Tolerance Host: if innate immunity is unable to eliminate *Mtb*, the host initiates adaptive immunity and granuloma formation, which is the beginning of the chronic phase of infection and disease tolerance to contain or ultimately eliminate *Mtb* (reverting host). Conditions associated with immunocompromised host may result in loss of *Mtb* containment and active disease in tolerant host. Although 90–95% of individuals are considered to be tolerant hosts, the exact number of these individuals who are able to clear *Mtb* or succumb to disease is still unknown. (3) Susceptible Host: individuals with impaired natural immunity to *Mtb* who progress to active disease and transmit the infection.

Exposure to *Mtb* either results in direct elimination of the pathogen, most likely by the innate immune system, or infection, and containment that requires both innate and adaptive immunity to form the granuloma (Figure 1). In 90–95% of individuals infected with *Mtb*, the bacteria are either eliminated or contained and remain in a latent state, termed latent tuberculosis infection (LTBI). These individuals are asymptomatic and do not transmit the disease (16). Both human and non-human primate (NHP) studies indicate that these asymptomatic LTBI individuals have a spectrum of infection that ranges from sterilized and well-contained infections to a small frequency of individuals who are at higher risk for reactivation (17–19). Although the mechanism(s) of host susceptibility to progressive disease is not well understood and is multifactorial, several genetic polymorphisms have been associated with risk of active TB. For instance, a type I IFN signature appears to be linked to development of active TB in NHP (20), and has been described as a marker of active TB in humans as well (21). This ultimately led to the discovery of extensive cellular and molecular mechanisms that were thought to be only engaged in host resistance to TB. However, recent studies indicate that some of these mechanisms, as detailed below, that were thought to be central to host resistance may also play an essential role in disease tolerance against *Mtb*.

## Granuloma is the Signature of Disease Tolerance in TB

Following the invasion of infectious agents (e.g., bacteria, fungi, and parasites), if the innate immune response is not able to destroy or expel the agent, the host will initiate an adaptive immune response. If the combination of both innate and adaptive immune responses fail to eliminate a pathogen, the host is then required to form granuloma—a mixture of both innate and adaptive effector cells—to “wall off” an agent and prevent dissemination. From that moment on, the host is forced to tolerate the agent. At the same time, this leads to a new set-point of immune responses with different magnitude as well as duration that must be carefully regulated to prevent immunopathology and maintain host fitness.

Granulomas are the hallmark of TB. However, they are a double-edged sword required for controlling and containment of *Mtb*, but also contribute to persistence of the bacteria (22–24). TB granulomas are particularly heterogeneous, but the basic granuloma architecture is composed of a central necrotic core (caseum), which is surrounded by mainly macrophages that are at different activation stages, and a cuff of T and B cells. Monocytes, neutrophils, DCs, and NK cells can also be found in the granulomas. The inflammatory state of granulomas can alter the ratio of its cellular composition, which becomes critical in determining granuloma fates and outcome of infection. Remarkably, despite being a critical step for the initial formation of the granuloma, it is still unclear if granuloma formation driven by the host or *Mtb*? While this fundamental question remains to be answered, tremendous advances in understanding the dynamics of granulomas in TB have recently been made.

While the induction of inflammatory mediators within a granuloma is required for preventing *Mtb* dissemination, overly intense pro-inflammatory responses lead to the destruction of granulomas via necrosis, enhanced lung parenchymal damage, lung cavitation, and transmission that results in the onset of active disease (25–27). Studies in animal models of TB as well as in humans have elegantly demonstrated that inflammatory signaling is highly organized in the granuloma as pro-inflammatory signaling is mainly found at the core of the granuloma, while anti-inflammatory signaling is located in the periphery (28). This spatial compartmentalization of pro- and anti-inflammatory signaling determines the granuloma's function in controlling bacterial dissemination. Thus, the host is better off with a balanced inflammatory and anti-inflammatory signaling that leads to the regulation of inflammation within and around the granuloma and reduced frequency of active disease (29).

## Mechanisms That Underlay the “Switch” From Host Resistance to Disease Tolerance

The central question that remains to be addressed is how and when host defense strategies switch from resistance to tolerance. While the exact cellular and molecular mechanisms of this phenomenon are still under investigation, we envision that three key pathways contribute to this transition.

### 1. Pathogen Recognition Signaling

During the early stage of infection, the vast majority of signaling in the host results from the detection of the pathogen that initiates predominantly anti-microbial host resistance to infection. Recognition of *Mtb* or mycobacterial products by pattern recognition receptors (PRRs) such as Toll-like receptors (TLRs), NOD-like receptors (NLRs), C-type lectin receptors (CLRs), and scavenger receptors initiates a cascade of events including production of cytokines, nitric oxide, reactive oxygen species, autophagy, and phagolysosome fusion to reduce the growth of *Mtb* and thus enhance host resistance (30). However, this initial host resistance to an infection comes with substantial tissue damage that needs to be repaired especially in a vital organ such as the lung. Additionally, in the context of a persistent infection like *Mtb*, in which innate immunity is often unable to eliminate the bacteria, controlling the magnitude of the inflammatory response becomes essential for host survival. Thus, as the infection persists, the host receives signals from damaged tissue to self-limit inflammation and preserve tissue integrity. For example, Mantovani's group has identified Toll/IL-1R (TIR) 8 receptor, a member of IL1R family, also known as single Ig IL-1-related receptor (SIGIRR), as a negative regulator of TLR/IL-1R signaling. TIR-8 signaling contributes to dampening inflammation and limiting tissue damage in *Mtb* infection (31). Mice deficient in *TIR-8* succumb to *Mtb* infection due to excessive inflammatory responses despite their ability to efficiently control bacterial growth (31). Further investigation is certainly required to dissect the pathways involved in regulating the inflammation to preserve tissue integrity and the maintenance of disease tolerance.

### 2. Host Immune Signaling

While the production of pro-inflammatory cytokines such as IL-1β and TNF-α are critical in anti-mycobacterial immunity predominantly during the early phase of *Mtb* infection, the constant production of these cytokines promotes inflammation-mediated tissue damage. Thus, their production needs to be tightly regulated. Sassetti's group has elegantly demonstrated that nitric oxide (NO) inhibits NLRP3 inflammasome-mediated IL-1β production to prevent neutrophil-dependent pulmonary tissue damage (32). Most recently, the same group has shown that the role of NO in host resistance to *Mtb* acts via the recruitment of neutrophils, which are permissive to *Mtb* growth (33). Importantly, this immunoregulatory function of NO is coordinated with the initial recruitment of IFN-γ-producing T cells into the lung, which leads to granuloma formation and perhaps the transition from host resistance to disease tolerance (please see the review from Sassetti-group in this special issue) (34).

The identification of mutations in the IL-12/IFN-γ/STAT1 axis that lead to disseminated mycobacterial infections, termed Mendelian Susceptibility to Mycobacterial Disease (MSMD), along with the susceptibility of T cell-deficient hosts to mycobacterial infections established the dogma that IFN-γ-producing T cells play a crucial role in host resistance against TB. However, there is no direct evidence of T cells/IFN-γ in protection against *Mtb*, but rather in the containment of infection (35–37) via regulation of the inflammatory response. For instance, extrapulmonary TB is associated with individuals having lower measurable Tuberculin Skin Test (TST) responses (38), as well as with HIV-positive individuals with very low CD4^+^ T cell counts (35). In addition, IFN-γ has been shown to inhibit pulmonary neutrophilic inflammation to prevent lung tissue damage during the chronic phase of *Mtb* infection (39, 40). High levels of neutrophils generate a strong inflammatory response that results in increased pulmonary pathology and mortality. Importantly, neutrophil depletion in IFNγR^−/−^ mice prolonged their survival during *Mtb* infection. (39). The contribution of neutrophils to immunopathology during *Mtb* infection has been well established in mice (41), NHP (42, 43), and humans (21). These studies collectively indicate that the IFN pathway is critical in the regulation of inflammatory signals and disease tolerance rather than host resistance.

Furthermore, dysregulated T cell responses appeared to be detrimental for the host by inducing overt immunopathology. It has been well documented that during chronic viral infection, constant exposure of T cells to antigens and inflammatory cytokines lead to loss of T cell function, a process termed “T cell exhaustion” (44). One of the well-defined pathways in T cell exhaustion is programmed cell death (PD1). The interaction between PD1, which is expressed on antigen-experienced T cells, and its ligands PDL-1 and PDL-2 prevents T cell proliferation and cytokine production. Thus, it was thought that the inhibition of PD1 signaling should promote protection via “reviving” T cell-mediated immunity to chronic *Mtb* infection. However, while disruption of PD1 signaling either genetically or via neutralizing antibodies significantly enhanced T cell-mediated immunity to *Mtb* infection, this was associated with increased bacterial growth, massive pulmonary immunopathology, and reduced survival (45, 46). Thus, the regulatory mechanisms involved in the expansion and contraction of T cell responses become a critical determinant of the outcome of TB infection. While the surface expression of some of these markers (e.g., PD1 or KLRG) on T cells appears to be critical for dictating their functional role during infection, the intrinsic immunoregulatory mechanisms of T cells are poorly understood.

Mitochondria are central platforms that critically regulate cell proliferation and differentiation. To meet the metabolic demands of active cells, mitochondria can rapidly switch from a state of catabolism to anabolism to provide the biosynthetic intermediates that are pivotal for cellular function. Naïve T cells have a low rate of metabolic activity, characterized by minimal nutrient uptake and biosynthesis. These cells procure cellular energy in the form of adenosine triphosphate (ATP) from the energetically efficient processes oxidative phosphorylation (OXPHOS) and fatty acid oxidation (FAO) (47). Upon TCR activation, dramatic metabolic reprogramming occurs to generate the increased energy needed for T cell proliferation, differentiation and cytokine production. To ensure adequate metabolic resources are available, activated T cells increase nutrient uptake and switch from OXPHOS and FAO to aerobic glycolysis (47). While energetically inefficient, glycolysis enables the cells to rapidly produce ATP and other biosynthetic precursors essential for cell growth and proliferation. This switch from predominantly OXPHOS to aerobic glycolysis, despite the presence of abundant oxygen, is known as the “Warburg Effect.” Metabolic shift from OXPHOS to glycolysis or vice-versa is also highly associated with the inflammatory and anti-inflammatory function of immune cells (48). For example, inflammatory cells such as activated macrophages exhibit higher glycolysis, by contrast anti-inflammatory cells such as M2 macrophages acquire higher OXPHOS than glycolysis (49). A recent study in non-human primates (NHP) suggests that the relative proportion of inflammatory or anti-inflammatory macrophages is important in deciding the outcome of *Mtb* infection (50). The metabolic status of a cell is also important to regulate immune cell polarization (51). Th17 cell differentiation relies on glycolysis, whereas blocking glycolysis inhibits Th17 development and promotes regulatory T cell (Treg) differentiation. Th17 cells are important in host resistance to *Mtb* but uncontrolled production of IL-17 induces inflammation via recruitment of neutrophils and increases the mortality of *Mtb*-infected mice (39). Higher susceptibility of TLR-2-KO mice to *Mtb* has been linked to reduced accumulation of Treg cells and concomitant increased inflammation (52). These findings suggest that the metabolic state determines the fate of immune cells which is critical in promoting or dampening inflammation.

An equally important function of mitochondria is their role in the cell death program. Cyclophilin D (CypD), a member of the cyclophilin protein family, is a conserved protein located in the mitochondrial matrix (53). It has been previously shown that CypD plays a key role in necrosis by regulating the mitochondrial permeability transition pore (MPTP), which allows the passage of solutes and water from the cytoplasm into the mitochondria (54, 55). Necrosis of macrophages is an exit mechanism for *Mtb* (56–59). Remold and colleagues initially demonstrated that the pharmacological inhibition of CypD in human macrophages lead to the inhibition of necrosis and reduction of *Mtb* growth *in vitro* (60). This observation has been recently extended to the zebrafish and mouse models of tuberculosis where the genetic blockade of CypD prevented macrophage necrosis and enhanced their anti-mycobacterial capacity (61, 62). Based on the role of CypD in macrophage immunity to *Mtb* infection, we initially hypothesized that CypD-deficient mice (CypD^−/−^) are resistant to *Mtb* infection. Surprisingly, CypD^−/−^ mice were highly susceptible to *Mtb* infection compared with control animals, despite similar numbers of bacteria in both groups. We further identified that this susceptibility was related to an enhanced T cell response that promoted lung immunopathology independent of host resistance. We have determined that CypD intrinsically regulates T cell metabolism and critically regulates disease tolerance in TB (63). Similarly, the C3HeB/FeJ mouse strain that generates a profound T cell response to *Mtb* infection quickly succumbs to death due to the overgrowth of necrotic granulomas (64, 65). Although we still don't know why the functional role of CypD is different in macrophages vs. T cells, we envision that as T cells are intrinsically programmed to proliferate, the functional role of CypD in these cells may be wired to regulate the metabolism and proliferation rather than cell death. Collectively, these data indicate that, similar to granulomas, T cells are a double-edged sword: while they are crucial to initiate granuloma formation during the early phase of *Mtb* infection and prevent the dissemination of disease, they also play an important role in transmission of *Mtb* by promoting granuloma necrosis during the active phase of the disease (66). Thus, the function and location of these effector cells are critical determinants of disease tolerance and host survival in TB.

### 3. Lung-Stromal Signaling

The term “tissue remodeling” refers to irreversible anatomical and structural changes. The lung injury caused by *Mtb* infection and subsequent granuloma formation results in distortion of the lung architecture. This requires effective and coordinated repair mechanisms to limit the extent of the granulomas and preserve lung function while ensuring pathogen containment. For instance, matrix metalloproteinases (MMPs), which are a family of zinc-dependent proteases, play an important role in extracellular matrix remodeling by degrading collagens. Several MMPs have been associated with active TB and cavitation (67), which reflects the importance of lung tissue repair in generating a preventive granuloma in TB. Furthermore, some of the mechanisms that are engaged in tissue healing, like fibrosis, also play a key role in the formation of fibrosis in the periphery of the granuloma to effectively prevent bacterial dissemination. Therefore, it is not surprising that the presence of type 2 immune responses, which are essential for controlling tissue damage, has commonly been observed in TB (68–71). While type 1 immune responses are crucial for the formation of an effective granuloma to control the infection, type 2 immunity is required at the same time to control lung tissue damages caused by both immune responses and *Mtb*. While the role of type 2 cytokines (e.g., IL-4 and IL-13) in stimulating TGF-β-dependent granulomatous inflammation and fibrosis is well established in parasitic infections, little is known about the exact role of these cytokines in tissue healing and repair in TB. During parasite infections both IL-4 and IL-13 are the major drivers of STAT6 translocation. STAT6-deficient mice are impaired in forming granulomatous fibrosis (72), and IL-13 increases TGF-β activation (73). Interestingly, using a heterologous mouse model of *Nippostrongylus brasiliensis* (Nb) and *Mtb* infection, Salgame's group has shown that the growth of bacteria was increased only at 4 weeks after *Mtb* infection, while there were no differences at two or seven weeks post infection. Despite this early increase in bacterial growth, there was no difference in lung histopathology or granuloma formation (74). Thus, while the type 2 immune bias transiently compromises early host resistance to *Mtb*, it may promote disease tolerance at later timepoints and ultimately control the infection. It therefore becomes important to identify the location of both innate and adaptive immune cells that are responsible for spatial production of type 1 and type 2 cytokines and extracellular matrix (ECM) remodeling in the granuloma.

Additionally, the expression of virulence factors from *Mtb* adds another layer of complexity for the maintenance of this delicate balance between host and *Mtb* in the granuloma. For instance, early secretory antigen-6 (ESAT6) appears to lyse lung epithelial cells and facilitate local dissemination (75). However, ESAT6 also induces MMP9 from epithelial cells, which was associated with the recruitment of monocytes/macrophages and granuloma maturation (76). In contrast to MMP9, it has been shown that MMP1 was significantly upregulated in individuals with active TB. MMP1 specifically degrades type I collagen and increases pulmonary tissue destruction in TB. Additionally, transgenic mice expressing human MMP1 showed extensive tissue damage despite similar levels of bacterial burden in the lungs (77). Interestingly, a recent study has reported that a selective MMP7 inhibitor (cipemastat) has a detrimental impact on pulmonary granulomas by increasing cavitation in a mouse model of TB (78). An elegant study by Tobin's group has also demonstrated extensive angiogenesis within the granuloma, whereas inhibition of vascular endothelial growth factor (VEGF) signaling reduced vascular leakage and bacterial dissemination in a zebrafish model of TB (79). Further studies also suggested that increased angiogenesis in the area that has restricted access to the blood supply may increase the access of immune cells and anti-TB drugs to the bacteria (80). Collectively these studies indicate that the location and balance in the signaling of type 1 and 2 immune responses that regulate lung extracellular matrix (ECM) remodeling via collagen deposition/degradation/angiogenesis define an effective granuloma in TB (please see the review from Tobin in this special issue).

## Conclusion and Remarks

A prolonged co-evolutionary interaction between humans and *Mtb* has almost reached its perfect balance with 90–95% of infected individuals being resilient or “tolerating” the presence of *Mtb* without any disease symptoms (Figure 1). This can be interpreted as 9 out of 10 people having a protective natural immunity against TB which renders them asymptomatic and non-infectious and may further explain why humans are the only known host for *Mtb* (14). This epidemiological data also suggests that, through a long evolutionary process, an equilibrium is reached that supports both host fitness and *Mtb* survival. Interestingly, in NHP which are the natural host for SIV, as well as in HIV-viremic pediatric and adult humans, it has been long recognized that viral replication is not the major cause of disease progression but rather immune cell activation (81–83). Similarly, reactivation of latent *Mtb* in a NHP coinfection model of SIV/*Mtb* was directly linked to over-activation of the immune response (84). Thus, it can be argued that the transition from HIV to AIDS, or LTBI to active TB may not depend on the pathogen load but rather on dysregulated immunity to infections.

While for an obvious reason we have been focusing on 5–10% of infected individuals who progress to active disease, we disproportionally have biased our scientific view as well as investigative approach toward resistance and the elimination of *Mtb*. Because of this bias, we incompletely understand the full spectrum of immunity to TB including the mechanisms of disease tolerance and thus fall short in developing an effective vaccine. Furthermore, any medical intervention targeting host resistance may potentially break disease tolerance which can have catastrophic consequences. While assessments of host resistance, in particular bacterial burden, is the gold standard for the evaluation of an effective therapy or vaccine, we propose that measurements of disease tolerance, such as immunopathology, are also important criteria to be considered in parallel to host resistance.

The new studies that shed light on disease tolerance may yield clinical benefit in designing host-targeted vaccines that minimize tissue damage, prevent granuloma cavitation and disease transmission, and ultimately reduce the global burden of TB disease.

## Author Contributions

MD wrote the manuscript with contributions from NK and EK.

### Conflict of Interest Statement

The authors declare that the research was conducted in the absence of any commercial or financial relationships that could be construed as a potential conflict of interest.
